# Deep-learning-based motion-correction algorithm in optical resolution photoacoustic microscopy

**DOI:** 10.1186/s42492-019-0022-9

**Published:** 2019-10-29

**Authors:** Xingxing Chen, Weizhi Qi, Lei Xi

**Affiliations:** 1 School of Electronic Science and Engineering, University of Electronic Science and Technology of China, Chengdu, 610054 Sichuan China; 2 Department of Biomedical Engineering, Southern University of Science and Technology, Shenzhen, 518055 Guangdong China

**Keywords:** Deep learning, Optical resolution photoacoustic microscopy, Motion correction

## Abstract

In this study, we propose a deep-learning-based method to correct motion artifacts in optical resolution photoacoustic microscopy (OR-PAM). The method is a convolutional neural network that establishes an end-to-end map from input raw data with motion artifacts to output corrected images. First, we performed simulation studies to evaluate the feasibility and effectiveness of the proposed method. Second, we employed this method to process images of rat brain vessels with multiple motion artifacts to evaluate its performance for in vivo applications. The results demonstrate that this method works well for both large blood vessels and capillary networks. In comparison with traditional methods, the proposed method in this study can be easily modified to satisfy different scenarios of motion corrections in OR-PAM by revising the training sets.

## Introduction

Optical resolution photoacoustic microscopy (OR-PAM) is a unique sub-category of photoacoustic imaging (PAI) [1–3]. Via the combination of sharp-focused pulsed laser and high-sensitivity detection of rapid thermal expansion-induced ultrasonic signals, OR-PAM offers both an optical-diffraction limited lateral resolution of micrometers and an imaging depth of millimeters. With these special features, OR-PAM is extensively employed in the studies of biology, medicine, and nanotechnology [4]. However, high-resolution imaging modalities are also extremely sensitive to motion artifacts, which are primarily attributed to the breath and heartbeat of animals. Motion artifacts are nearly inevitable for imaging in vivo targets, which cause a loss of key information for the quantitative analysis of images. Therefore, the exploration of image-processing methods that can reduce the influence of motion artifacts in OR-PAM is necessary.

Recently, several motion-correction methods have been proposed for PAI to obtain high-quality images [5–8]. The majority of existing algorithms are primarily based on deblurring methods that are extensively employed in photoacoustic-computed tomography (PACT) and only suitable for cross-sectional B-scan images [5, 6]. Schwarz et al. [7] proposed an algorithm to correct motion artifacts between adjacent B-scan images for acoustic-resolution photoacoustic microscopy (AR-PAM). Unfortunately, the algorithm needs a dynamic reference, which is not feasible in high-resolution OR-PAM images. A method presented by Zhao et al. [8] has the capability of addressing these shortcomings but can only correct the dislocations along the direction of a slow-scanning axis. Recent methods that are based on deep learning have demonstrated a state-of-the-art performance in many fields, such as natural language processing, audio recognition and visual recognition [9–14]. Deep learning discovers an intricate structure by using a backpropagation algorithm to indicate how a net should change its internal parameters, which are used to compute the representation in each layer from that in the previous layer. A convolutional neural network (CNN) is a common model for deep learning in image processing [15]. In this study, we present a fully CNN [16] to correct motion artifacts in a maximum amplitude projection (MAP) image of OR-PAM instead of a volume. To evaluate the performance of this method, we conduct both simulation tests and in vivo experiments. The experimental results indicated that the presented method can eliminate displacements in both simulations and in vivo MAP images.

## Methods

### Experimental setup

The OR-PAM system in this study has been described in previous publications [17]. A high-repetition-rate laser serves as an irradiation source with a repetition rate of 50 KHz. A laser beam is coupled into a single mode fiber, collimated via a fiber collimation lens (F240FC-532, Thorlabs Inc.), and focused by an objective lens to illuminate a sample. A customized micro-electro-mechanical system scanner is driven by a multifunctional data acquisition card (PCI-6733, National Instrument Inc.) to realize fast raster scanning. We detect photoacoustic signals using a flat ultrasonic transducer with a center frequency of 10 MHz and a bandwidth of 80% (XMS-310-B, Olympus NDT). The original photoacoustic signals are amplified by a homemade pre-amplifier at ~ 64 dB and digitized by a high-speed data acquisition card at a sampling rate of 250 MS/s (ATS-9325, Alazar Inc.). The imaging reconstruction is performed using Matlab (2014a, MathWorks). We derived the envelopes of each depth-resolved photoacoustic signal using the Hilbert transform and projected the maximum amplitude along the axial direction to form a MAP image. We implemented our algorithm for motion correction using a tensor flow package and trained this neural network using Python software on a personal computer.

### Algorithm of CNN

**Fig. 1:**
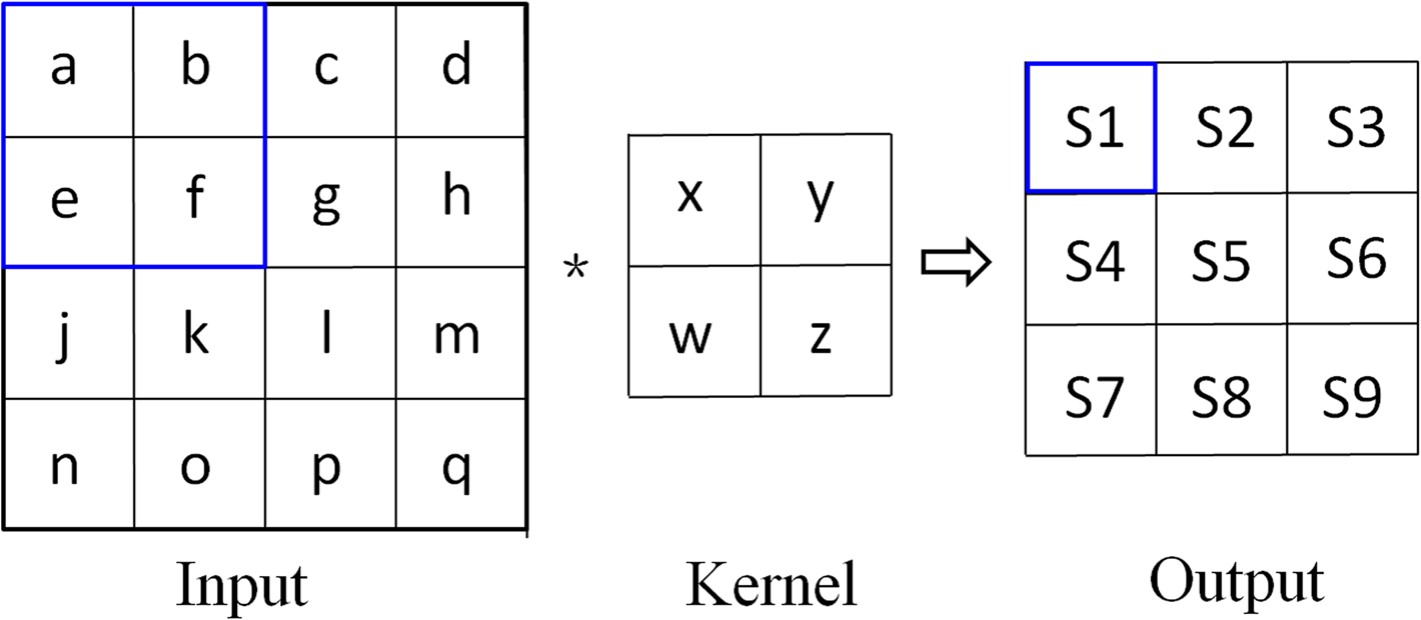
Mapping processes of convolutional neural network

Figure 1 illustrates an example of the mapping processes of CNN. In this case, the input is a two-dimensional 4 × 4 matrix, and the convolution kernel is a 2 × 2 matrix. First, we select four adjacent elements (a, b, e, f) in the upper right corner of the input matrix, multiply each element with the corresponding element in the convolution kernel, and sum all calculated elements to form S1 in the output matrix. We repeat the same procedure by shifting the 4 × 4 matrix by one pixel in either direction of the input matrix to calculate the remaining pixel values in the output matrix. The CNN is classified by two major properties: local connectivity and parameter sharing. As depicted in Fig. 1, the element S1 is not associated with all elements in the input layer; it is only associated with a small number of elements in a spatially localized region (a, b, e, f). A hidden layer has several feature maps, and all hidden elements within a feature map share the same parameter, which further reduces the number of parameters.

**Fig. 2:**
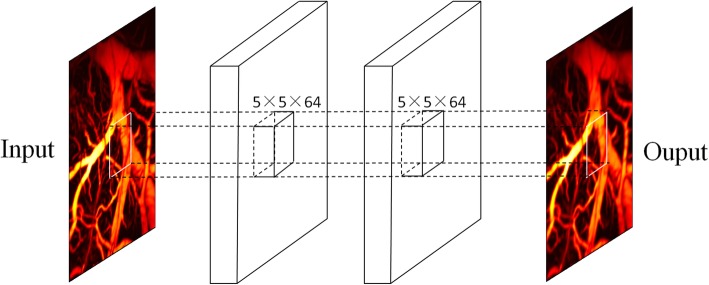
Structure of motion correction based on convolutional neural network

The structure of the CNN in this work is illustrated in Fig. 2. The images with the motion artifacts used for training were obtained from the ground-truth image. As depicted in Fig. 2, the method consists of three convolutional layers. The first convolutional layer can be expressed as

1 $$ {\mathbf{G}}_{\mathbf{1}}=\mathbf{Relu}\left({\mathbf{W}}_{\mathbf{1}}\ast \mathbf{I}+{\mathbf{B}}_{\mathbf{1}}\right) $$

where the rectified linear unit (Relu) is a nonlinear function max(0, z) [18], W_1_ is the convolution nucleus, ∗ denotes the convolution operation, I is the original image, and B_1_ is the neuron bias vector. The second convolutional layer, which is a nonlinear mapping, can be defined as

2 $$ {\mathbf{G}}_{\mathbf{2}}=\mathbf{Relu}\left({\mathbf{W}}_{\mathbf{2}}\ast {\mathbf{G}}_{\mathbf{1}}+{\mathbf{B}}_{\mathbf{2}}\right) $$

where Relu, W_2_, B_2_, and ∗ are defined according to the previously defined expression. In comparison with the first two layers, a nonlinear function does not exist in the last layer, which is used to reconstruct the output image. The last layer can be defined as follows:

3 $$ \mathbf{O}=\left({\mathbf{W}}_{\mathbf{3}}\ast {\mathbf{G}}_{\mathbf{2}}+{\mathbf{B}}_{\mathbf{3}}\right) $$

Similarly, W_3_ and B_3_ are defined according to the previously defined expression. In this study, the input and output images have one channel; thus, the size of the convolution nucleus W_1_, W_2_, and W_3_ are set to [5, 5, 1, 64], [5, 5, 64, 64], and [5, 5, 64, 1], respectively. The size of the neuron bias vectors B_1_, B_2_, and B_3_ are set to [64], [64], and [1], respectively.

### Training

Learning the end-to-end mapping function M requires estimation of the network parameters Φ = { W_1_, W_2_, W_3_, B_1_, B_2_, B_3_ }. The purpose of the training process is to estimate and optimize the parameters W_1_, W_2_, W_3_, B_1_, B_2_, and B_3_, which is achieved by minimizing the error between the reconstructed images M(O; Φ) and the corresponding input images I. Given a set of motion images and their corresponding non-motion images, we use the mean squared error as the loss function:

4 $$ \mathbf{L}\left(\boldsymbol{\Phi} \right)=\frac{\mathbf{1}}{\boldsymbol{n}}{\sum}_{\boldsymbol{i}=\mathbf{1}}^{\boldsymbol{n}}{\left\Vert \mathbf{M}\left({\mathbf{O}}_{\boldsymbol{i}};\boldsymbol{\Phi} \right)-{\mathbf{I}}_{\boldsymbol{i}}\right\Vert}^{\mathbf{2}} $$

where *n* is the number of training samples. The error is minimized using the gradient descent with standard backpropagation [19]. To avoid changing the image size, all convolutional layers are set to the same padding.

## Results

**Fig. 3:**
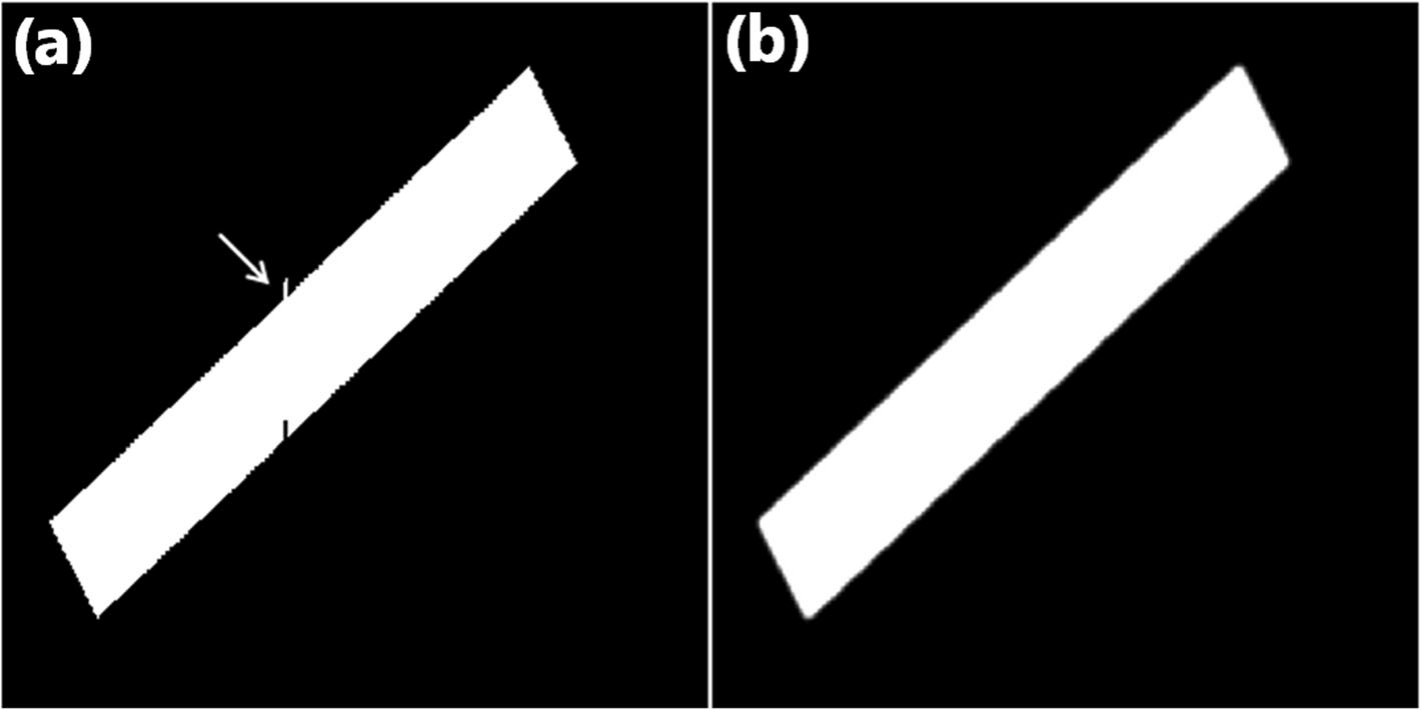
Results of simulation experiment

After the training, we conducted a series of experiments to evaluate the performance of the method. In the simulation, we created a displacement along the direction of the Y axis, which is denoted by a white arrow (Fig. 3(a)). We processed the image with the trained CNN and obtained the results, as depicted in Fig. 3(b). In comparison with the images before and after the processing, we observe that the displacement has been corrected, which demonstrates that our algorithm works well in simulation cases.

**Fig. 4:**
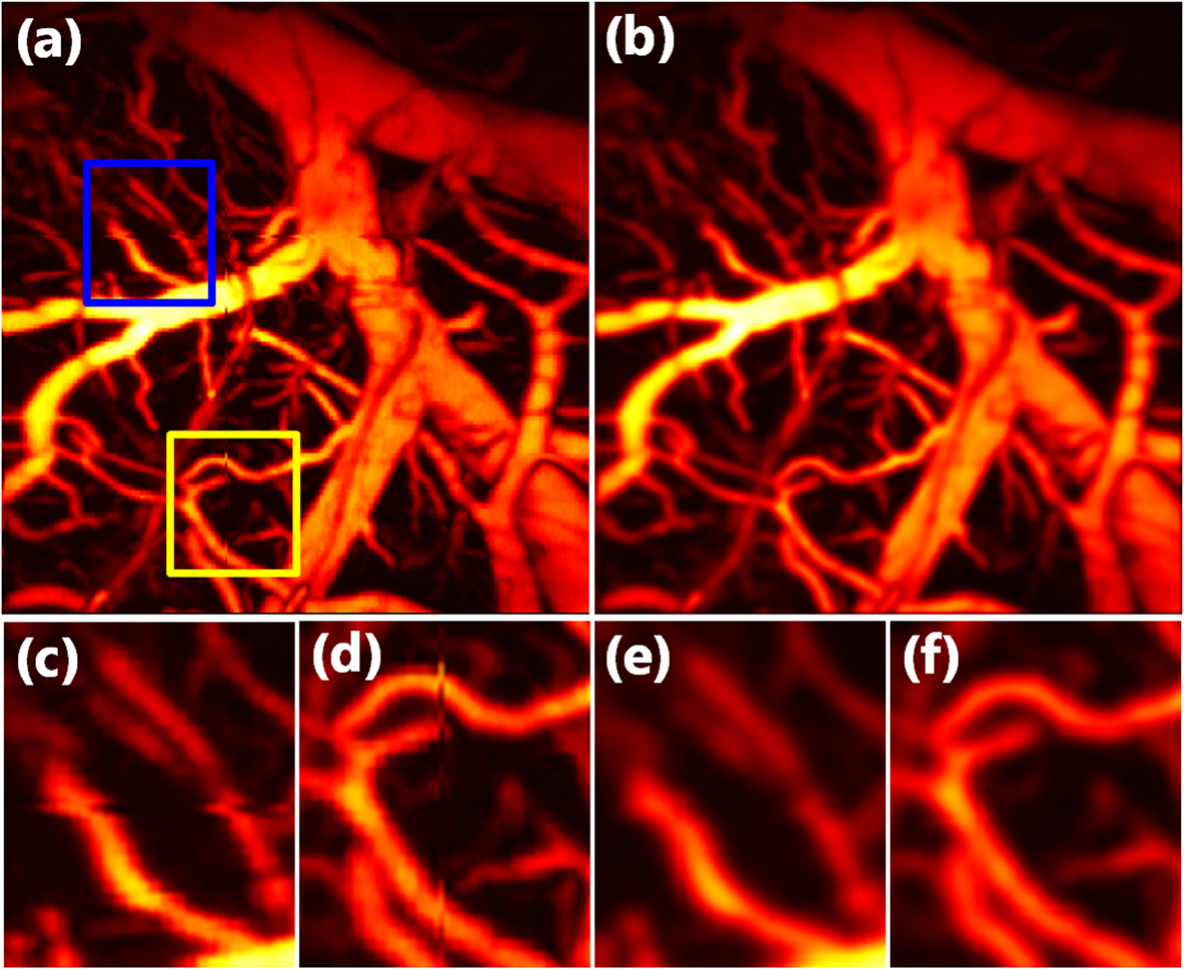
Results of correcting motion artifacts in horizontal and vertical dislocation. **a** MAP image that corresponds to the raw data of a rat brain. **b** MAP image after motion correction. **c** and **d** Enlarged images of the two boxes in (**a**). **e** and **f** Enlarged figures of corresponding areas in (**b**)

We created both horizontal artifacts and vertical motion artifacts, as depicted in Fig. 4(a). Figure 4(c) and (d) illustrate an enlarged view of the motion artifacts in the blue rectangle and yellow rectangle, respectively. Figure 4(b) depicts the corrected MAP image via the proposed method, in which both the horizontal artifact and the vertical motion artifact have been corrected, as depicted in Fig. 4(e) and Fig. 4(f).

**Fig. 5:**
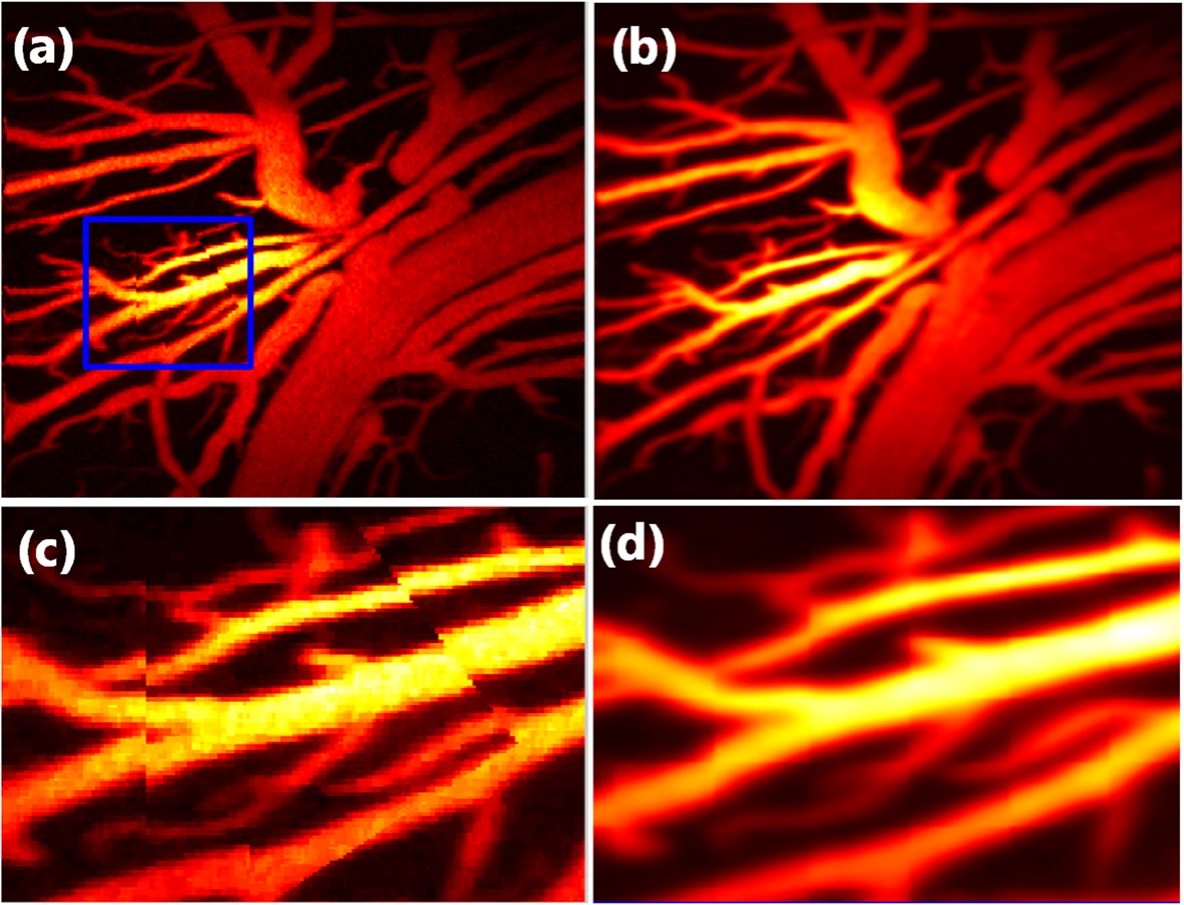
Results of correcting motion artifacts in an arbitrary dislocation. **a** Maximum amplitude projection (MAP) image that corresponds to the raw data of a rat brain. **b** MAP image after motion correction. **c** Enlarged image of the box in (**a**). **d** Enlarged figure of corresponding areas in (**b**)

To demonstrate that our method can adequately correct motion artifacts in an arbitrary direction, we established two complicated motion artifacts, as depicted in Fig. 5(a) and (c). Figure 5(b) and (d) illustrate the corrected MAP images, in which both displacements in the vertical and tilted directions have been corrected.

**Fig. 6:**
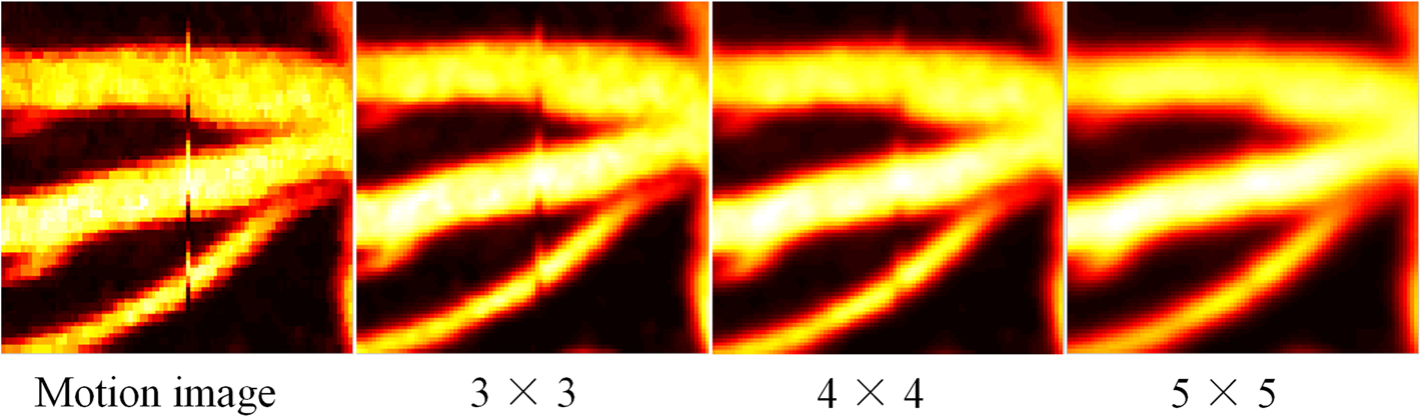
Results using different kernel sizes

We evaluated the network performance using different kernel sizes. We conduct three experiments: (1) the kernel size in the first experiment has a size of 3 × 3; (2) the kernel size in the second one has a size of 4 × 4; and (3) the kernel size in the third experiment has a size of 5 × 5. The results in Fig. 6 suggest that the performance of this algorithm can be significantly improved by using a larger kernel size. However, the processing efficiency will decrease. Thus, the choice of the network scale should always be a trade-off between performance and speed.

## Conclusions

We experimentally demonstrated the feasibility of the proposed method using a CNN to correct motion artifacts in OR-PAM. In comparison with the existing algorithms [5–8], the proposed method demonstrates a better performance in eliminating motion artifacts in all directions without any reference objects. Additionally, we verified that the performance of the method improves as the kernel size increases. Although this method is designed for OR-PAM, it is capable of correcting motion artifacts in other imaging modalities, such as photoacoustic tomography, AR-PAM, and optical coherence tomography, when the corresponding training sets are used.

## Data Availability

The datasets generated and/or analyzed during the current study are not publicly available due to personal privacy but are available from the corresponding author on reasonable request.

## Abbreviations

AR-PAM: Acoustic-resolution photoacoustic microscopy
CNN: Convolutional neural network
MAP: Maximum amplitude projection
OR-PAM: Optical-resolution photoacoustic microscopy
PAI: Photoacoustic imaging
